# Urine Output as a Novel Predictor for In-Hospital Mortality in Acute Pulmonary Embolism Patients: Training With the MIMIC Database and Validation With Independent Cohort

**DOI:** 10.1155/cdr/7907049

**Published:** 2025-02-26

**Authors:** Wenjia Ai, Fangfei Li, Qilin Yang, Jingluo Qiu, Aiqiang Zhou, Yinqian Huang, Baohui Xu, Zhihui Zhang

**Affiliations:** ^1^Department of Vascular Surgery, The Second Affiliated Hospital, Guangzhou Medical University, Guangzhou, China; ^2^Department of Surgery, Stanford University School of Medicine, Stanford, California, USA; ^3^Department of Rheumatology, Guangzhou First People's Hospital, South China University of Technology, Guangzhou, China; ^4^Department of Critical Care, The Second Affiliated Hospital of Guangzhou Medical University, Guangzhou, China

**Keywords:** acute pulmonary embolism, Cox regression, in-hospital mortality, risk factor, urine output

## Abstract

**Background:** Identifying high-risk patients with acute pulmonary embolism is vital for improving disease prognosis. However, current guidelines and research on risk factors are insufficient to meet clinical needs. This study was aimed at exploring novel risk factors to predict in-hospital mortality.

**Methods:** We utilized a patient cohort from the Medical Information Mart for Intensive Care Version IV (MIMIC-IV) database as training cohort. Major analyses included screening risk factors for in-hospital mortality, correlation analysis via smooth curve fitting, multivariate Cox regression, and subgroup analysis. The findings were further validated with our own institute patient cohort.

**Results:** Among 1463 adult patients with acute pulmonary embolism in the MIMIC-IV database, the overall in-hospital mortality rate was 17.8%. A nonlinear correlation was observed between urine output and in-hospital mortality. A urine discharge less than 0.85 mL/kg/h was used as the threshold and was negatively associated with the risk for in-hospital death. Compared to patients with urine value < 0.5 mL/kg/h, the risk for in-hospital mortality reduced by 36% and 48% in patients with urine values of 0.5–0.85 mL/kg/h and > 0.85 mL/kg/h, with the hazard ratios of 0.64 (0.47, 0.87) and 0.52 (0.38, 0.72), respectively. This association remained significant in the subgroup analysis after adjusting for age, gender, hypotension, and low oxygen saturation. Our validation patient cohort (*n* = 151) further confirmed the strong association of the urine value with in-hospital mortality and consistent cutoff value.

**Conclusion:** Our study revealed a negative association of urine output with in-hospital mortality in acute pulmonary embolism patients, with the optimal urine output being significantly higher than the value of other critical illnesses.

## 1. Introduction

Acute pulmonary embolism (APE) is a significant cause of mortality in hospital settings [1]. Clinical trials have emphasized the importance of promptly identifying high-risk cases of pulmonary embolism, with the increased rates of both mortality and morbidity [2]. Current practice guidelines such as the 2019 European Society of Cardiology recommend the use of pulmonary embolism severity index (PESI), which is estimated based on factors including systolic blood pressure less than 100 mmHg, age more than 80 years, chronic obstructive pulmonary disease, malignancy, heart rate exceeding 110 bpm, and arterial oxyhemoglobin saturation less than 90% [3]. Moreover, the PESI is a simplified parameter and is informative for clinical prediction applications [4]. However, both underprediction and refinement of the index need to be improved. According to a multi–US center survey, the PESI, simple PESI, and Bova score risk stratification, all of these are only able to predict the risk of 7-day and 30-day mortality moderately and slightly, respectively [5]. Yu et al. reported enhanced prognostic prediction for pulmonary embolism by correlating subgroup (age, heart rate, and partial pressure of arterial oxygen) analyses [6]. Stratified analysis [7] revealed that a change in the heart rate threshold from 110 to 140 bpm increased the specificity of the Bova score from 93.2% to 98.0% among patients at moderate and high risk. There is also a demand for more accurate novel clinical markers to identify high risk patients who need more aggressive treatments such as reperfusion or thrombectomy for better clinical outcomes [8–11].

Urine output is commonly monitored in the intensive care unit as a prognostic indicator [12]. Reduced urine output was associated with increased hospital mortality in critically ill patients [13, 14]. However, the complexity of severely ill patients as well as additional factors that influence mortality and urine output must be considered [15]. Additionally, patients with APE often experience right heart dysfunction which potentially impacts fluid balance and urine output [16].

In this study, the APE patient cohort from the Medical Information Mart for Intensive Care Version IV (MIMIC-IV) database was used as the training cohort. We screened the risk factors for in-hospital mortality through correlation analysis using smooth curve fitting, multivariate Cox regression, and subgroup analysis. A nonlinear correlation was observed between urine output and in-hospital mortality, with a threshold of 0.85 mL/kg/h indicating that the minimum optimal urine output in APE patients is significantly higher than that in other critical illnesses. A urine output below this threshold was negatively associated with the risk for in-hospital mortality. Additionally, the findings from the training cohort were validated using the patient cohort from our institute.

## 2. Materials and Methods

### 2.1. Participants and Data Source

We performed a retrospective cohort study of patients with APE in the MIMIC-IV database which contains patients' clinical information in the intensive care unit at the Beth Israel Deaconess Medical Center [17]. These patients served as our training cohort. Furthermore, eligible patients were retrieved from the Second Affiliated Hospital of Guangzhou Medical University electronic medical record database from 2013 to 2023 and used as our validation cohort. Guidelines for reinforcing the reporting of observational epidemiology studies for observational cohort studies were followed. This study was approved by the Second Affiliated Hospital of Guangzhou Medical University Ethics Committee.

### 2.2. Study Population and Variable Extraction

#### 2.2.1. Patient Databases

**Figure 1:**
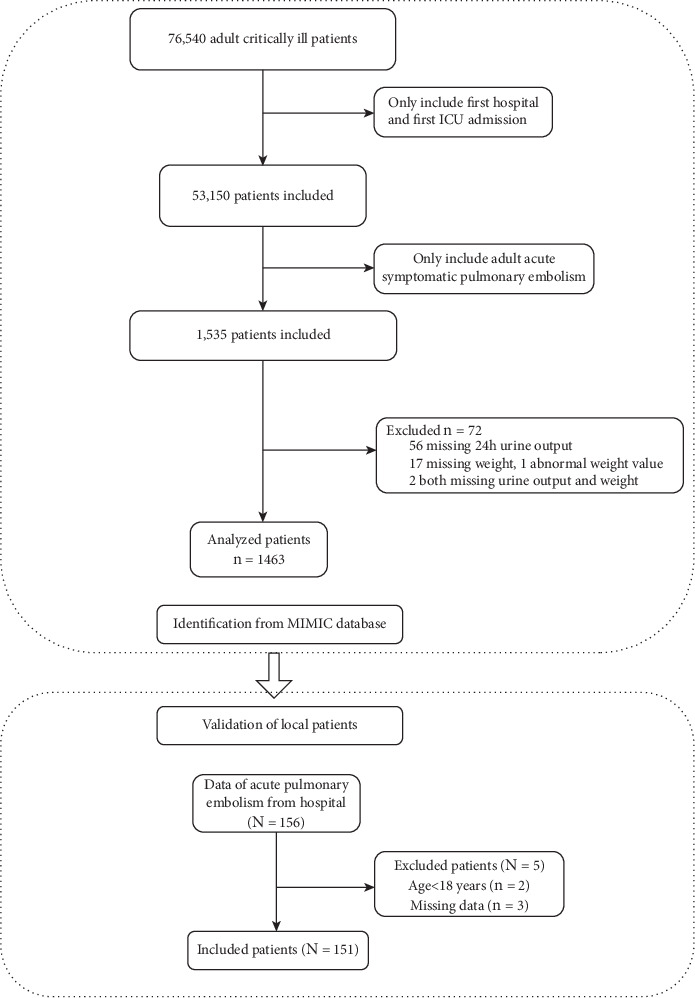
Flow diagram for patient screening and enrollment in MIMIC-IV training and our own validation cohorts.

The MIMIC-IV database was publicly available (https://physionet.org/content/mimiciv/0.4/). The principles of inclusion were adult patients (≥ 18 years) with APE, defined as International Classification of Diseases (ICD)-9 (41511, 41512, 41519, and 67382) or ICD-10 (12609) code and admitted to the intensive care unit (Figure 1). We excluded patients with medical histories of pulmonary embolism, pulmonary artery hypertension, or sepsis. We adopted the first intensive care unit admission date only for patients admitted to the intensive care unit more than once. Validation of the hospital database was performed from our institute. The inclusion criteria were adult patients (≥ 18 years) with APE using ICD-9 or codes as those for patients in the MIMIC database. Patients who did not receive therapeutic anticoagulation after diagnosis and had a history of pulmonary embolism were excluded.

#### 2.2.2. Variable Collection

Urine output was recorded as the total volume (milliliter) excreted during the first 24 h in the intensive care unit. Additional variables, based on our clinical experience and published literature, were sex, age, body weight, systolic blood pressure, heart rate, oxygenated hemoglobin saturation, cancer, dialysis history, use of renal replacement therapy on Day 1, and comorbidities (chronic pulmonary disease, congestive heart failure, peripheral vascular, cerebrovascular, and liver dysfunction).

#### 2.2.3. Outcome

In-hospital mortality, which was defined as death from any cause before hospital discharge, was the primary outcome.

### 2.3. Statistical Analysis

#### 2.3.1. Baseline Characteristics of Patients

For continuous variables, the data are presented as mean and standard deviation (SD) when normally distributed and median and interquartile range (IQR) if not normally distributed. Categorical variables were given as the percentage. The chi-square test, one-way ANOVA, and Kruskal–Wallis tests were used to determine the statistical significance among the groups for categorical, normally distributed, or nonnormally distributed variables, accordingly. Smooth curve fitting, multivariate Cox regression analyses, and subgroup analyses were employed to evaluate the independent relationship between in-hospital mortality and risk factors.

All analyses were carried out using Free Statistics software version 1.7 and the statistical software packages R 3.3.2 (http://www.r-project.org, The R Foundation). The hypothesis tests were two-sided, with a significance level of 0.05.

## 3. Results

### 3.1. Patient Characteristics

Among 76,540 adult critically ill patients in the MIMIC-IV database, the final training cohort consisted of 1463 consecutive patients with APE according to the ICD definition and missing data (Figure 1). Nearly 50% of the patients were male, aged 65.5 ± 16.2 years. The total in-hospital mortality rate was 17.8%, and these patients (median age: 69.2 years) were slightly older than those who survived (64.7 years). In univariate analysis, age, systolic blood pressure, heart rate, oxygenated hemoglobin saturation, respiratory rate, cancer diagnosis, peripheral vascular disease, cerebrovascular disease, blood urea nitrogen, and urine output were statistically significantly different between survivors and nonsurvivors. Admission urine output was significantly higher in survivors (0.8 ± 0.6 mL/kg/h) than that in nonsurvivors (0.6 ± 0.6 mL/kg/h) (Table 1, *p* < 0.001).

The validation cohort consisted of 156 consecutive patients from our institute, with five patients that were excluded due to missing data (Figure 1). The overall in-hospital mortality rate was 14.6%, with an average age of 60.6 ± 17.4 years and 76 males. In univariate analysis, heart rate, oxygenated hemoglobin saturation, and urine output were statistically significantly different between survivors and nonsurvivors. The admission urine output was higher in survivors (0.9 ± 0.5 mL/kg/h) than in nonsurvivors (1.4 ± 0.4 mL/kg/h) (Table 2, *p* < 0.001).

### 3.2. Nonlinear Relationship Between Urine Output and In-Hospital Mortality in the Training Patient Database

**Figure 2:**
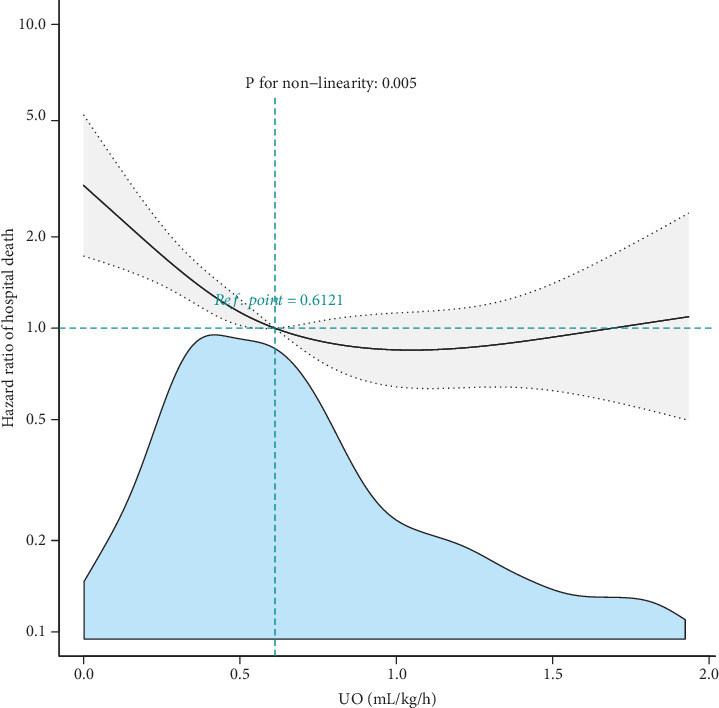
Smooth curve fitting of the relationship between baseline admission urine output and in-hospital mortality. Solid and dashed lines represent the estimated values and their corresponding 95% confidence intervals. Adjusted for gender, age, systolic blood pressure, heart rate, cancer, dialysis, blood urea nitrogen, peripheral vascular disease, cerebrovascular disease, chronic obstructive pulmonary disease, and blood oxygen saturation.

A smooth curve-fitting model adjusted for major confounders, including sex, age, systolic blood pressure, heart rate, blood oxygen saturation, cancer, dialysis, blood urea nitrogen, peripheral vascular disease, cerebrovascular disease, and chronic obstructive pulmonary disease, revealed a nonlinear correlation between urine output and in-hospital mortality (Figure 2, *p* = 0.005). In further inflection point analysis, we identified a critical threshold of approximately 0.85 mL/kg/h (95% confidence interval (CI) of 0.82–0.87) for urine output. Below this threshold, urine output was negatively correlated with in-hospital mortality. This threshold was apparently higher than that in most critically ill patients.

### 3.3. Relationship Between Urine Output and In-Hospital Mortality in the Training APE Patient Database by Trend Test

According to a piecewise multivariate Cox regression model, a significant association between urine output and in-hospital mortality (*p* for trend, Table 3) was found even after being adjusted for major confounding factors, including gender, age, systolic blood pressure, heart rate, blood oxygen saturation, and comorbidities. Specifically, compared to individuals with urine output less than 0.5 mL/kg/h, individuals with a urine output of 0.5–0.85 mL/kg/h had a 36% reduction in the risk for in-hospital death (hazard ratio (HR): 0.64 with the 95% CI of 0.47–0.87), while those with a urine output exceeding 0.85 mL/kg/h had a 48% reduction the risk for in-hospital death (HR: 0.52 with the 95% CI of 0.38–0.72).

### 3.4. Subgroup Analysis of the Relationship Between In-Hospital Mortality and Urine Output in the Training Patient Database

**Figure 3:**
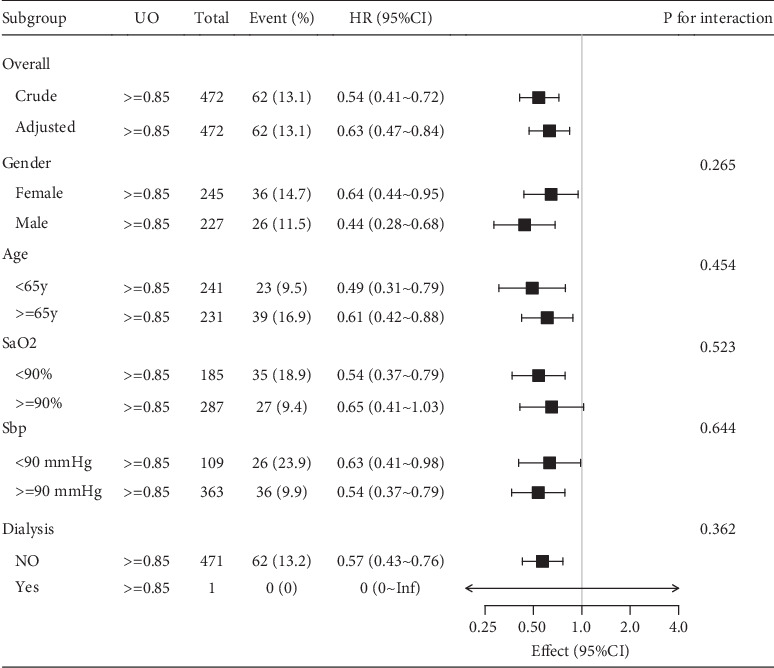
Subgroup analyses and interaction analyses of association of urine output with in-hospital mortality of the MIMIC-IV database.

There was a 37% reduction in the risk for in-hospital mortality in patients with a urine output exceeding 0.85 mL/kg/h (HR: 0.63 with the 95% CI of 0.47–0.84; Figure 3) compared to those with a urine output < 0.85 mL/kg/h. This association was still present in the subgroup analysis stratified by age, sex, and even hypotension or low oxygen saturation. Subgroup analyses were also performed based on the presence of confounders, including weight, systolic blood pressure, heart rate, cancer, peripheral vascular disease, cerebrovascular disease, and chronic obstructive pulmonary disease. However, this association was not detected in the subgroup analysis of patients undergoing dialysis.

### 3.5. Association of Urine Output With the Risk for In-Hospital Mortality in the Validation Patient Database

**Figure 4:**
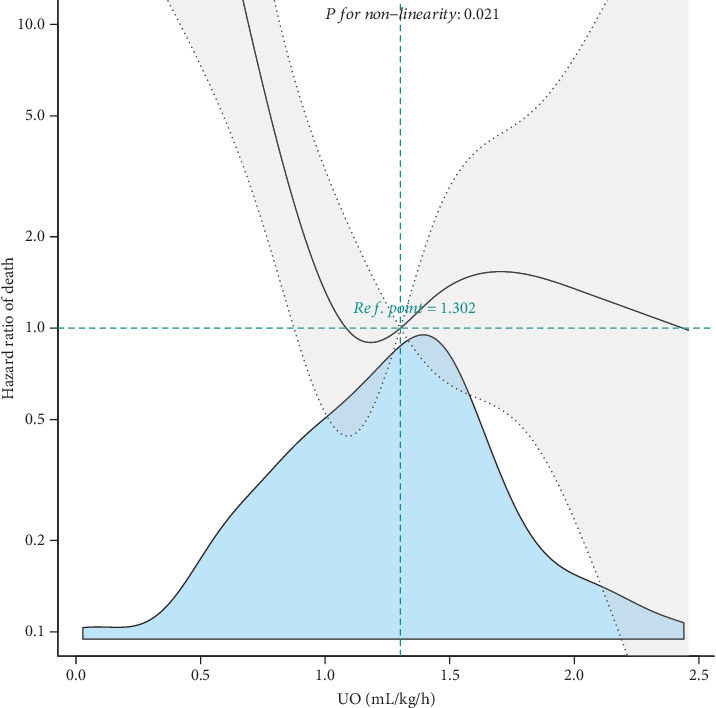
Smooth curve fitting of the relationship between admission urine output and in-hospital mortality for validated participants. Solid and dashed lines represent the estimated values and their corresponding 95% confidence intervals. Adjusted for gender, age, systolic blood pressure, heart rate, cancer, dialysis, blood urea nitrogen, chronic pulmonary disease, and blood oxygen saturation.

To validate the findings in the training cohorts, we performed a similar analysis using independent patient cohorts built from our institute patient electronic medical record database (Figure 1). In our validation cohort, as demonstrated by a smooth curve-fitting model, we confirmed a nonlinear relationship between urine output and in-hospital mortality. The threshold for urine output was 0.82 mL/kg/h, with a 95% CI of 0.80–0.84, which approximated the value in the MIMIC training patient cohort (0.85 mL/kg/h). Urine output was also negatively associated with the risk for in-hospital death in the validation patient cohort (Figure 4). In the multivariate Cox regression model, patients with a urine output exceeding 0.85 mL/kg/h had an 89.5% reduction in the risk for in-hospital death (HR: 0.10 with a 95% CI of 0.04–0.28) in relative to patients with a urine output of < 0.85 mL/kg/h (Table 4). All these findings were consistent with the findings in the MIMIC training patient database.

## 4. Discussion

In this retrospective cohort study, we observed a negative correlation between urine output and the risk for in-hospital mortality in patients with APE in both training and independent validation cohorts. This association remained significant even after being adjusted for major risk factors including demographic factors. Exceeding the baseline urine output threshold of 0.85 mL/kg/h was found to reduce the risk for in-hospital mortality. Furthermore, our own validation cohort showed a highly consistent association between urine output and in-hospital mortality. Our study highlights the significance of monitoring admission urine output in APE patients, particularly those with a urine output below the specified cutoff value.

We found distinct baseline characteristics in surviving and nonsurviving patients in the MIMIC training patient database, including heart rate, systolic blood pressure, respiratory rate, cancer, oxygenated hemoglobin saturation, and urine output. It has been reported that urine output is associated with adverse outcomes in patients with atherosclerotic cardiovascular disease and all-cause mortality [18, 19]. In an analysis of 3458 patients with chronic liver disease, the addition of urine output to the diagnostic criteria increased the measured incidence of acute kidney failure compared with the use of the creatinine criteria alone [20]. This approach is beneficial for identifying high-risk patients. However, this was not observed in other diseases, including trauma and postsurgical morbidities [21–23]. This discrepancy may have resulted from high heterogeneity in urine output thresholds among admission diagnoses. Thus, further analysis is needed to avoid inappropriate dichotomization across the cohort and to accurately identify patient-specific thresholds.

Tatlisu et al. reported that a high blood urea nitrogen level of 34.5 mg/dL at admission predicted all-cause in-hospital mortality among patients with APE. They found a significantly greater in-hospital mortality rate of 51.1% in the group with higher blood urea nitrogen levels compared to the group with lower blood urea nitrogen levels (1.9%) [24]. In another study, patients with creatinine levels of 50–100 *μ*mol/L and a urine output of > 1 mL/kg/h had a lower mortality rate (12%) than patients with creatinine levels of > 200 mol/L and a urine output of 1 mL/kg/h (20%). Interestingly, there was no significant variance in urine output thresholds among patients with routine creatinine levels [15]. Our study revealed that the optimal urine output threshold was approximately 0.85 mL/kg/h. This was greater than the urine output threshold of 0.5 mL/kg/h, which is commonly used as an indicator of acute kidney injury and a higher mortality rate [25]. However, in the MIMIC database analysis, neither multivariate Cox regression nor subgroup analysis revealed an association between blood urea nitrogen levels and the risk for in-hospital mortality. This finding was further confirmed in our independent validation patient cohort analysis.

Main APE pathology is increased pulmonary artery pressure due to vasoconstriction or occlusion, which consequently leads to right ventricular dysfunction and right heart congestion, the former dysfunction impairing cardiac output and ultimately causing prerenal oliguria [16]. Fluid overload due to renal venous congestion may also contribute to renal dysfunction [26]. Therefore, elevated urine output may compensate for patient recovery. Conversely, excessive urine output may result in fluid imbalance, electrolyte disturbances, and immunosuppression [27]. In a multicenter, randomized, controlled trial, restrictive fluid management lowered adverse events in critically ill patients with acute kidney injury [28]. The minimum threshold of urine output in our study was greater than that in other critical illnesses, which may indicate that urine output in patients with pulmonary embolism is more strongly associated with multiorgan function and has a greater impact on prognosis. Future research should investigate the balance between fluid control and urine output in critically APE patients.

This study has several limitations. First, due to the observational study design, we cannot draw any causal inferences. Second, additional confounding factors including the underlying cause of APE, renal insufficiency, and its medication were not sufficiently documented in either the training or validation patient cohorts due to its retrospective nature; thus, we cannot rule out the impact of these factors, although major risk factors have been adjusted for. Third, we considered the minimum ideal urine output for patients with critical APE, and future studies should include individual factors such as renal insufficiency and fluid intake in the data analysis. Finally, the effectiveness of diuretic therapy in oliguric patients with APE remains uncertain based on this retrospective study. Thus, further prospective studies should focus on the impact of preload and diuretic treatment.

## 5. Conclusion

Our study demonstrated a negative association between urine output and the risk of in-hospital mortality in patients with APE, underscoring the higher optimal cutoff value and potential application in predicting clinical outcome.

## Figures and Tables

**Table 1:** Patient characteristics in MIMIC-IV database by survival and hospital death.

**Characteristic**	**Total cases (1463)**	**Survival cases (1202)**	**Death (261)**	**p** ** value**
Sex (male, *n*, %)	727 (49.7)	602 (50.1)	125 (47.9)	0.521
Age (mean ± SD)	65.5 ± 16.2	64.7 ± 16.5	69.2 ± 14.1	< 0.001
Signs				
Systolic blood pressure (mmHg, mean ± SD)	92.3 ± 17.3	94.2 ± 16.1	83.7 ± 19.5	< 0.001
Heart rate (beats/minute, mean ± SD)	91.8 ± 17.1	91.0 ± 16.5	95.4 ± 19.3	< 0.001
SaO_2_ (%, median and interquartile)	96.3 (94.8, 97.7)	96.3 (94.9, 97.7)	95.8 (94.4, 97.7)	0.006
Respiratory rate (breaths/minute, mean ± SD)	30.0 ± 6.9	29.6 ± 6.9	31.4 ± 7.0	< 0.001
Urine output (mL/kg/hour, mean ± SD)	0.8 ± 0.6	0.8 ± 0.6	0.6 ± 0.6	< 0.001
Medical history				
Diabetes (*n*, %)	282 (19.3)	225 (18.7)	57 (21.8)	0.247
Congestive heart failure (*n*, %)	363 (24.8)	287 (23.9)	76 (29.1)	0.076
Peripheral vascular disease (*n*, %)	103 (7.0)	73 (6.1)	30 (11.5)	0.002
Cerebrovascular disease (*n*, %)	205 (14.0)	150 (12.5)	55 (21.1)	< 0.001
Chronic obstructive pulmonary disease (*n*, %)	426 (29.1)	351 (29.2)	75 (28.7)	0.881
Cancer (*n*, %)	395 (27.0)	297 (24.7)	98 (37.5)	< 0.001
Dialysis (*n*, %)	32 (2.2)	18 (1.5)	14 (5.4)	< 0.001
Blood urea nitrogen (mg/dL, median and interquartile)	19.0 (14.0, 28.8)	18.0 (13.0, 26.0)	27.0 (18.0, 42.0)	< 0.001

*Note:p* value for the comparison between survival and death groups.

Abbreviations: MIMIC-IV: Medical Information Mart for Intensive Care Version IV. SaO_2_: oxygenated hemoglobin saturation. SD: standard deviation.

**Table 2:** Patient characteristics in the validation cohort by survival and hospital death.

**Characteristic**	**Total cases (** **n** = 151**)**	**Survival cases (** **n** = 129**)**	**Death cases (** **n** = 22**)**	**p** ** value**
Sex (male, *n*, %)	76 (50.3)	66 (51.2)	10 (45.5)	0.621
Age (years, mean ± SD)	60.6 ± 17.4	60.1 ± 17.5	63.7 ± 16.6	0.365
Signs				
Systolic blood pressure (mmHg, mean ± SD)	111.3 ± 17.9	111.7 ± 17.7	109.3 ± 18.9	0.570
Heart rate (beats/minute, mean ± SD)	98.9 ± 21.8	96.8 ± 20.6	110.8 ± 25.0	0.005
SaO_2_ (%, median and interquartile)	96.0 (92.0, 98.0)	96.5 (93.0, 98.0)	94.5 (80.5, 97.0)	0.002
Respiratory rate (breaths/minute, mean ± SD)	23.0 ± 5.1	22.7 ± 4.8	24.5 ± 6.1	0.138
Urine output (mL/kg/hour, mean ± SD)	1.3 ± 0.5	1.4 ± 0.4	0.9 ± 0.5	< 0.001
Past medical history				
Hypertension (*n*, %)	54 (37.0)	42 (33.9)	12 (54.5)	0.064
Diabetes (*n*, %)	27 (18.5)	21 (16.9)	6 (27.3)	0.246
Cognitive heart failure (*n*, (%))	39 (25.8)	36 (27.9)	3 (13.6)	0.158
Peripheral vascular disease (*n*, (%))	19 (12.6)	17 (13.2)	2 (9.1)	0.741
Cerebral vascular disease (*n*, %)	32 (21.2)	28 (21.7)	4 (18.2)	1
Chronic obstructive pulmonary disease (*n*, %)	25 (17.0)	21 (16.8)	4 (18.2)	1
Cancer (*n*, %)	32 (21.2)	24 (18.6)	8 (36.4)	0.087
Dialysis (*n*, %)	2 (1.3)	1 (0.8)	1 (4.5)	0.273
Blood urea nitrogen (mg/dL, mean ± SD)	5.8 (4.0, 7.7)	5.4 (3.9, 7.6)	6.6 (5.3, 8.0)	0.751

*Note:p* value for the comparison between survival and death groups.

Abbreviations: SaO_2_: oxygenated hemoglobin saturation. SD: standard deviation.

**Table 3:** Multivariate Cox regression for the association of admission urine output with in-hospital mortality in patients from the MIMIC-IV database.

**Variable**	**Not adjusted**		**Minimally adjusted model**		**Fully adjusted model**	
	**HR (95% CI)**	**p** ** value**	**HR (95% CI)**	**p** ** value**	**HR (95% CI)**	**p** ** value**
Urine output	0.45 (0.34~0.59)	< 0.001	0.48 (0.36~0.63)	< 0.001	0.58 (0.45~0.76)	< 0.001
Quartiles						
< 0.5	1 (Ref)		1 (Ref)		1 (Ref)	
0.5–0.85	0.48 (0.36~0.65)	< 0.001	0.49 (0.36~0.66)	< 0.001	0.64 (0.47~0.87)	0.004
≥ 0.85	0.40 (0.29~0.54)	< 0.001	0.43 (0.32~0.59)	< 0.001	0.52 (0.38~0.72)	< 0.001
Test for the trend	0.61 (0.52~0.71)	< 0.001	0.64 (0.54~0.74)	< 0.001	0.71 (0.61~0.84)	< 0.001

*Note:* Minimally adjusted model: age and sex. Fully adjusted model: age, sex, systolic blood pressure, heart rate, cancer, dialysis, blood urea nitrogen, peripheral vascular disease, cerebrovascular disease, chronic obstructive pulmonary disease, and blood oxygen saturation.

Abbreviations: CI: confidence interval. HR: hazard ratio.

**Table 4:** Multivariate Cox regression of the association of admission urine output with in-hospital mortality in the validation patient cohort.

**Urine output threshold**	**Cases**	**HR (95% CI)**	**p** ** value**
< 0.85 mL/kg/h	26	1 (Ref)	
≥ 0.85 mL/kg/h	125	0.11 (0.03~0.35)	0.0002

*Note:* Fully adjusted model: age, sex, systolic blood pressure, heart rate, cancer, dialysis, blood urea nitrogen, peripheral vascular disease, cerebrovascular disease, chronic obstructive pulmonary disease, and blood oxygen saturation.

Abbreviations: CI: confidence interval. HR: hazard ratio.

## Data Availability

The MIMIC-IV database training dataset was downloaded from a public database at the website https://mimic.physionet.org. The validation patient dataset is available from the corresponding author upon reasonable request.

## Nomenclature

APE: acute pulmonary embolism
ICD: International Classification of Diseases
MIMIC-IV: Medical Information Mart for Intensive Care Version IV
